# How Microglia Manages Non-cell Autonomous Vicious Cycling of Aβ Toxicity in the Pathogenesis of AD

**DOI:** 10.3389/fnmol.2020.593724

**Published:** 2020-11-17

**Authors:** YunHee Seol, Soomin Ki, Hannah L. Ryu, Sooyoung Chung, Junghee Lee, Hoon Ryu

**Affiliations:** ^1^Center for Neuroscience, Brain Science Institute, Korea Institute of Science and Technology, Seoul, South Korea; ^2^Department of Brain and Cognitive Science, Ewha Womens University, Seoul, South Korea; ^3^Department of Neurology, Boston University Alzheimer’s Disease Center, Boston University School of Medicine, Boston, MA, United States; ^4^VA Boston Healthcare System, Boston, MA, United States

**Keywords:** Alzheimer’s disease, microglia, amyloid-β, non-cell-autonomous toxicity, vicious cycle

## Abstract

Alzheimer’s disease (AD) is a progressive neurodegenerative disease and a common form of dementia that affects cognition and memory mostly in aged people. AD pathology is characterized by the accumulation of β-amyloid (Aβ) senile plaques and the neurofibrillary tangles of phosphorylated tau, resulting in cell damage and neurodegeneration. The extracellular deposition of Aβ is regarded as an important pathological marker and a principal-agent of neurodegeneration. However, the exact mechanism of Aβ-mediated pathogenesis is not fully understood yet. Recently, a growing body of evidence provides novel insights on the major role of microglia and its non-cell-autonomous cycling of Aβ toxicity. Hence, this article provides a comprehensive overview of microglia as a significant player in uncovering the underlying disease mechanisms of AD.

## Introduction

Alzheimer’s disease (AD) is the most common form of dementia, with increasing prevalence as global life expectancy increases. The aggregation of β-amyloid (Aβ), the main component of senile plaques, is a pathological hallmark in AD. In the context of abnormal Aβ processing, ineffective Aβ phagocytosis and clearance by non-neuronal cells including microglia and astrocytes are linked to AD pathogenesis, but the exact mechanism is not understood yet. Microglia, the resident macrophages of the brain, constantly survey the microenvironment for pathogen-associated (PAMPs) or damage-associated molecular patterns (DAMPs), and clear unwanted toxins for steady-brain maintenance (Janeway, 1992; Kigerl et al., 2014; Clayton et al., 2017). As such, microglia have been widely viewed as a homogenous population of ancillary cells, which nevertheless become neurotoxic under neurodegenerative conditions (Lee et al., 2016).

Recent studies have yielded new interpretations of the involvement of microglia in Aβ pathogenesis. Comprehensive single-cell RNA analysis of brain immune cells revealed a disease-associated reactive microglia phenotype called DAM (disease-associated reactive microglia). Owing to the microglial receptors TREM2 (triggering receptor expressed on myeloid cells 2) and Tyrobp (Tyro protein tyrosine kinase binding protein), DAM cells exhibit remarkable morphological changes polarized from homeostatic microglia in response to Aβ (Clayton et al., 2017). Additionally, in studies of the brain immune system regarding pyrin domain-containing protein 3 (NLRP3) positive (+) inflammasomes, ASC Specks (Apoptosis-associated speck-like protein) containing a C-terminal caspase recruitment domain (CARD), and Aβ-ASC composites, the view upon microgliosis or the secondary cellular responses to Aβ pathology have been increased.

Previous studies report that microglia undergo prominent morphological and functional changes with a marked decline in homeostatic signatures in AD (Krasemann et al., 2017; Mathys et al., 2017; Zhou et al., 2020). Hypothetically, cross Aβ aggregation (so-called cross-seeding activity) of different amyloid proteins *via* a synergetic relationship between non-neuronal cells may contribute to neuronal injury. Contrary to previous dogma, microglia are not merely subsidiary to the disease mechanisms of AD. Accordingly, in this review, we revisit the pathway of Aβ aggregation and focus on microglia function in non-cell-autonomous pathways of sustained Aβ-dependent pathogenesis in AD.

## Birth of Amyloid-Beta Peptide

### Amyloid Precursor Protein (APP) Synthesis and Trafficking

**Figure 1:**
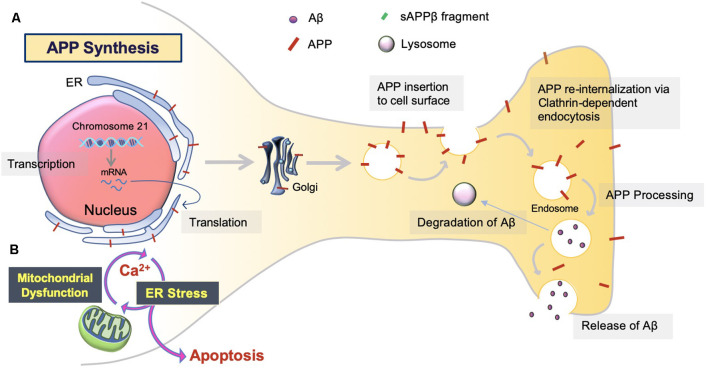
Amyloid precursor protein (APP) synthesis and trafficking. **(A)** APP synthesis. APP synthesis commences with transcription on chromosome 21. Then, it is translated by endoplasmic reticulum (ER) bound ribosomes, translocated into the ER, and trafficked to the Golgi to assume native structure and folding (Sun et al., 2015; Sole-Domenech et al., 2016). Eventually, the APP is inserted into the plasma membrane and reinternalized to produce Aβ. Otherwise, APP can be redirected to non-amyloidogenic pathways by the retromer complex and transported back to the Golgi or plasma membrane *via* the recycling pathway. Nascent Aβ peptides are either extracellularly released or degraded by the lysosome (Hernández-Zimbrón and Rivas-Arancibia, 2014). **(B)** ER stress. Aβ toxicity responds with consequential disruption of Ca^2+^ permeability *via* mitochondria-associated membrane (MAM), mitochondrial Ca^2+^ uptake, and eventual apoptosis.

**Figure 2:**
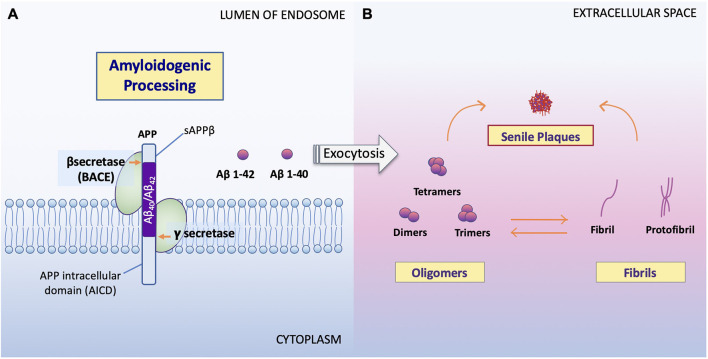
Processing of APP and formation of Aβ oligomers vs. fibrils. **(A)** Amyloidogenic processing. Sequence divergence at the internal Aβ site by γ-secretase produces the monomers Aβ1–40 and Aβ1–42 (Campion et al., 2016) to be deposited to the extracellular space. **(B)** Aβ oligomers vs. fibrils formation. Amyloid oligomers are small with few-chain, soluble, disordered clusters. Fibrils are long, many-chained, highly structured, β-sheet-like aggregates. The hydrophobic amino acids of Aβ1–40 and Aβ1–42 peptides confer to the hydrophobicity of the C terminal. Fibrils then arrange in a beta-pleated sheet and form the amyloid plaques seen on conventional hematoxylin and eosin (HE) staining or amyloid specific staining (Sun et al., 2015). It has been noted that the high peptide concentration tips the balance towards fibrils regardless of monomer concentrations and that the onset of fibrilization limits the concentration of oligomers in solution (Schmit et al., 2011).

The overproduction and extracellular deposition of Aβ1–42 peptides by neurons is a major component of Aβ senile plaque formation and maturation (Lee and Ryu, 2010; Schmit et al., 2011; Campion et al., 2016; Daria et al., 2017; Picone et al., 2020). How Aβ accumulates in the brains of the elderly individual is unclear, but could be initiated by changes in amyloid precursor protein (APP) metabolism (Figure 1). Under normal conditions, APP synthesis continues at a high rate in which a portion of APP proteins are inserted into subcellular organelles *via* the early secretory pathway (Anelli and Sitia, 2008; Campion et al., 2016). APP is transported and inserted into the plasma membrane, where more than 90%; is cleaved non-amyloidogenically by α-secretase and γ-secretase activity (Hernández-Zimbrón and Rivas-Arancibia, 2014; Sole-Domenech et al., 2016). APP is also re-internalized and directed to late endosomes in a clathrin-dependent manner where it is cleaved by β-secretase (BACE1) at the N-terminal and γ-secretase at the C terminal (Hernández-Zimbrón and Rivas-Arancibia, 2014; Sun et al., 2015; Webers et al., 2020). Sequence divergence at the internal Aβ site generates Aβ1–40, Aβ1–42, and a long-form of APP (sAPPβ) fated to dispatch into the extracellular space. As well as the amino-terminal APP intracellular domain (AICD) which is released into the cytosol (Figure 2; Campion et al., 2016).

Studies show that soluble Aβ oligomers or small Aβ aggregates are toxic to neurons in AD (Jarrett et al., 1993; Sun et al., 2015; Picone et al., 2020), whereas Aβ fibril formation has been proposed as a neural-protective process, possibly segregating neurons from soluble Aβ (Sun et al., 2015; Campion et al., 2016; Picone et al., 2020). Indeed, now, it is well accepted that Aβ toxicity, which disrupts neuronal cell structures and cellular function, resulting in cell death, is mainly represented by oligomers or small aggregates (Daria et al., 2017; Reiss et al., 2018; Picone et al., 2020).

Notably, previous studies show evidence of Aβ associated toxicity in larger Aβ species or plaques that are surrounded by microglia (Sheng et al., 2019; Dickson et al., 1999; Serrano-Pozo et al., 2016). Interestingly, microglia surround Aβ plaques and exhibit decreased Aβ clearance in late-stage AD (Condello et al., 2015; Yuan et al., 2016; Webers et al., 2020). Furtheri nvestigation into the various forms of Aβ depositions and its relationship to non-cell-autonomous mechanisms are necessary for understanding exactly how microglia engage in Aβ clearance and how they are responsible for Aβ accumulation (Sole-Domenech et al., 2016).

### Endoplasmic Reticulum (ER) Stress

Considering only 10%; of APP is inserted into the plasma membrane, APP processing may not be isolated to late-endosomes (Figures 1, 2). For instance, Aβ and β-secretases have been identified in cellular compartments involved in APP processing, recycling, and degradation (Picone et al., 2020). One such area of interest is the endoplasmic reticulum (ER; Hashimoto and Saido, 2018). Increased levels of unfolded protein response (UPR) in AD post mortem brain tissues, perhaps with the retention of Aβ or APP in the ER lumen, have been described (Hoozemans et al., 2005, 2009; Nijholt et al., 2012; Picone et al., 2020). Aβ oligomers activate mitochondrion-ER stress-mediated apoptosis, in which, a special sub-compartment called the mitochondria-associated membrane (MAM) is responsible for Ca^2+^ homeostasis. Subsequently, Ca^2+^ uptake due to exogenous Aβ results in eventual apoptosis, promoting the release of Aβ and Ca^2+^ into the extracellular space and triggering inflammatory responses (Resende et al., 2008; Song et al., 2008; Costa et al., 2010; Plácido et al., 2014; Picone et al., 2020). The role of ER stress in AD is still poorly understood. Therefore, more investigation is necessary to determine the Aβ-associated neuronal damage along this cell-autonomous pathway (Plácido et al., 2014; Hashimoto and Saido, 2018).

## Which Cell Type Works on Amyloid-Beta Clearance?

### Homeostatic Microglia

**Figure 3:**
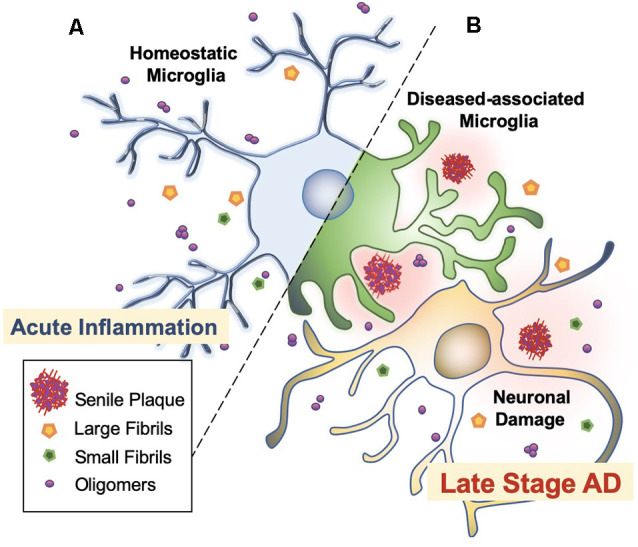
Microglial activation checkpoints. **(A)** Homeostatic microglia. Resting microglia, under non-inflammatory healthy conditions, have a small soma with processes extending into the microenvironment (Webers et al., 2020). Triggering of PAMP or DAMP signals may occur intermittently, in which ramified microglia exhibit “controlled phagocytosis,” a process by which cells engage in the clearance of damaged cells and debris as part of their scavenging role within the brain (Sole-Domenech et al., 2016; Webers et al., 2020). **(B)** Disease-associated microglia. Intermediate activation of microglia exhibits larger and elongated soma, where microglia become highly motile with enhanced phagocytic activity and release pro-inflammatory cytokines (O’Brien and Wong, 2011). Fully activated microglia are located nearby Aβ plaques. In AD transgenic mice, gene set enrichment analysis revealed significant involvement in phagocytotic pathways, endocytosis, and regulation of anti-inflammatory and immune response in DAM (Keren-Shaul et al., 2017).

Microglia are extremely sensitive resident myeloid cells of the central nervous system (CNS; Anwar and Rivest, 2020). Microglia play a variety of roles to clear dying neurons, proteins, and debris *via* active phagocytosis and micropinocytosis activity (Rogers et al., 2002; Webers et al., 2020). Phagocytosis by microglia is the process of “eating” large unwanted or toxic macromolecules that are delivered to the lysosome to break down materials, similar to the autophagy process in neurons (Malik et al., 2019; van Weering and Scheper, 2019; Anwar and Rivest, 2020). This includes the clearance of both soluble and insoluble Aβ peptides in the healthy brain, preventing Aβ over-accumulation, and thus, preempting AD initiation (Anwar and Rivest, 2020; Webers et al., 2020). However, in the majority of AD cases, it is unclear whether the faster production or slower clearance of Aβ species is responsible for plaque accumulation (Sole-Domenech et al., 2016). It has been reported that microglia sufficiently clear Aβ in the early stages of AD while late-stage AD is characterized by the phagocytosis of fibrils and plaques with an overflow of Aβ in the brain (Figure 3; Hickman et al., 2008; O’Brien and Wong, 2011; Keren-Shaul et al., 2017; Anwar and Rivest, 2020; Webers et al., 2020). A shortage of protein clearance by microglia may ultimately swing the balance between neuronal health and death (Zhao et al., 2017; Malik et al., 2019). Thus, the progression of AD may strongly depend on microglial phagocytosis and autophagy-lysosomal activity. In this paradigm, the connection between phagocytosis and microglia-mediated neurotoxicity is closely linked to the pathogenesis of AD (O’Brien and Wong, 2011).

### Disease-Associated Reactive Microglia (DAM)

**Figure 4:**
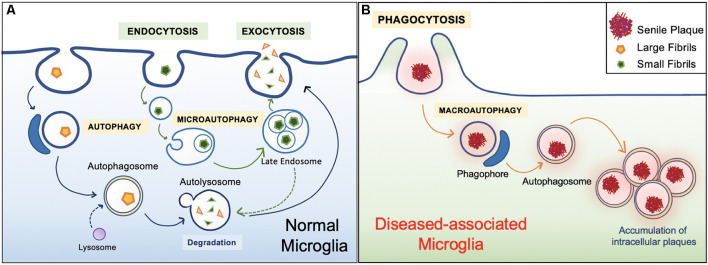
Healthy vs. diseased Aβ clearance. **(A)** Homeostatic autophagy. In the healthy brain, endocytosis of Aβ oligomers and fibrils are normal (Lee and Landreth, 2010; Webers et al., 2020). Internalized Aβ substrates are sorted within the endocytic pathway. Aβ substrates are delivered to acidic lysosomes and late endosomes. Compartment acidification leads to degradation of Aβ oligomers and fibrils, then released *via* exocytosis (Sole-Domenech et al., 2016). **(B)** Diseased Aβ clearance. In the unhealthy brain, microglia engulf large Aβ plaques *via* phagocytosis. However, Aβ plaques are not properly degraded and instead harbored within the cell, ultimately leading to inflammatory conditions (Malik et al., 2019; Zhao and Zhang, 2019).

In general, the healthy hippocampal parenchyma is characterized by an even distribution of microglia. However, in human cases and animal models of AD, microglia in the vicinity of Aβ deposits lose their Aβ clearing ability (Figure 4). IBA1 positive (+) cells express an accumulation of autophagy receptor p62 in the late stages of AD (O’Brien and Wong, 2011; Daria et al., 2017; Tejera et al., 2019; Anwar and Rivest, 2020). This phenomenon has been attributed to a specific subpopulation of microglial cells. Microglia in *5xFAD* mice and human post mortem AD brains transit from a homeostatic phenotype to DAM population, accompanied by alterations to their morphology and gene transcription (O’Brien and Wong, 2011; Sole-Domenech et al., 2016; Keren-Shaul et al., 2017; Lučìūnaitė et al., 2019). Immunohistochemical staining also revealed the DAM as autophagosomes with positively stained intracellular Aβ (Keren-Shaul et al., 2017).

Two activation states of microglia have been defined: Stage I and Stage II DAM (Da Mesquita and Kipnis, 2017). Importantly, Aβ1–42 has been identified as one of the damage-associated molecular patterns (DAMPs) that triggers the microglial transformation (Cho et al., 2014; Terrill-Usery et al., 2014; Deczkowska et al., 2018). In its diseased state, microglia express a single-pass transmembrane receptor (*Trem2*) which associates with the signaling adaptor tyrosine kinase-binding protein (Tyrobp). Studies have indicated the role of Trem2 in phagocytosis along the activation spectrum that generates DAM in microglia (Kleinberger et al., 2014). In a study by Ulland et al., increased LC3^+^ puncta, an autophagosome marker, was observed in human post-mortem brain sections from AD patients with a rare R47H variant of the Trem2 gene compared to controls (Ulland et al., 2017; Filipello et al., 2018; Ulland and Colonna, 2018). In *Trem2*^−^/*5xFAD* mice, similar results were observed in addition to the failure of microglia to migrate around Aβ plaques compared to controls (Kleinberger et al., 2014; Ulland et al., 2017; Ulland and Colonna, 2018). Furthermore, Keren-Shaul et al. demonstrated an absence of full DAM in *Trem2*^−^/5xFAD mice, instead, a large population of partially activated or Stage I DAM was accumulated in the brain, indicating that DAM activation occurs in a TREM2-independent manner (Haure-Mirande et al., 2019). Whereas, entering Stage II DAM required the activation of Tyrobp in a TREM2-dependent manner (Keren-Shaul et al., 2017). It is well known that in both mouse and AD patient brains, Tyrobp is significantly upregulated (Ma et al., 2015). Mice expressing a decrease in Tyrobp protein resulted in impaired microglial activation decreased microglial recruitment and clustering around Aβ plaques (Haure-Mirande et al., 2017). This may indicate an enhanced phagocytotic role of Tyrobp in close association with Trem2, however, its role remains elusive. Overall, homeostatic microglia phenotype from Stage I and then Stage II DAM is accompanied by pronounced DAM-specific gene expression with full microglial activation (Keren-Shaul et al., 2017). The loss of function or partial defect in the trajectory of microglial activation is likely to accumulate and facilitate in the development of AD.

Such findings coincide with Johnson et al.’s (2020) recent large-scale proteomic study uncovering the cellular changes complementary to AD. A consensus view of the proteomic changes within each progressive AD state was developed using a co-expression analysis and a weighted correlation network analysis algorithm (WGCNA). Interestingly, the co-expression module with the most strongly altered AD proteins linked to AD genetic risk was the Astrocyte/Microglia metabolism module, which was enriched in proteins linked to microglia, astrocytes, and sugar metabolism. Specifically, its principal component (PC) was more strongly associated with the neuropathological hallmarks of AD (CERAD, Braak-staging-system, MMSE, CDR) compared to other biological processes (mitochondria, RNA binding/splicing). Importantly, in this module, the microglia protein markers were identified to be in an anti-inflammatory disease-associated state. Synonymous with current studies on microglia activation, it also exhibited one of the strongest increasing patterns along with AD progression in the dorsolateral prefrontal cortex (DLPC; Johnson et al., 2020). It is noticeable that most phenotypic markers amongst a thousand AD risk factor genes were categorized into an anti-inflammatory phagocytotic DAM state. Thus, the strong AD correlations with DAM highlights the importance of heterogeneous microglia populations in the brain. Microglia may be characterized by a heterogeneous pool with local and cross-seeding effects on Aβ clearance (Mandrekar-Colucci and Landreth, 2010). On this note, depletion of either old or young microglial cells prevented Aβ plaque clearance in AD brains, indicating the modulation effect of old microglial populations by young microglia (Malik et al., 2019). Furthermore, Daria et al. (2017) found that co-cultured organotypic brain slices of amyloid bearing APP/PS1 transgenic AD mice with the brain slices of young neonatal wild-type mice revealed almost a 60%; reduction in Aβ plaque levels. After microglia were depleted of clodronate in young brain slices and then added to old APP/PS1 mouse brain slices, the clearance of plaques was less effective. Moreover, young microglia-derived conditioned media increased the proliferation of old microglia and decreased the size of Aβ plaques. This study suggests that microglia activity can be reversibly regulated and that microglial aging is an instrumental factor in Aβ plaque phagocytosis and clearance (Daria et al., 2017).

### Phagocytosis and Autophagy of Aβ

Recent studies suggest a “critical threshold” of microglial capacity or limitation in Aβ degradation (Anwar and Rivest, 2020; Pomilio et al., 2020). Pomilio et al. (2020) monitored the autophagic flux and inflammation of microglia in AD. Prolonged inflammatory responses or persistent exposure to Aβ1–42 peptides and fibrillar Aβ resulted in microglial exhaustion and decreased autophagy markers. In this case, short vs. long exposure of Aβ on microglia (*in vitro* cultured BV-2 cells) showed significant and differential changes in autophagy activity. Short (2 h) exposure of Aβ1–42 peptide exhibited functional autophagy and enhanced autophagic flux in an inflammasome-mediated manner. However, prolonged Aβ1–42 exposure (longer than 24 h) resulted in a decreased autophagic flux. Together, aggregated Aβ peptides affect the autophagy flux in microglia in a time-dependent manner (Pomilio et al., 2020). Consequently, such microglial autophagy dysfunction enhances toxic Aβ protein aggregates, possibly leading to neuronal damage in AD patients (Figure 3; Anwar and Rivest, 2020).

### Lysosomal Damage

In microglia, internalized Aβ substances are delivered to the lysosome for degradation (Nakanishi, 2003; Halle et al., 2008; Ries and Sastre, 2016). This interplay between autophagy machinery and lysosomal activity has been considered in the context of chronic Aβ exposure (Zhang et al., 2017). Lysosomal damage was associated with autophagic impairment and membrane permeabilization of acid hydrolase cathepsin-D, which altered LysoTracker staining in the cytoplasm of microglia. Furthermore, microglia in the vicinity of amyloid deposits in post-mortem AD brains showed phagocytic CD69 positive (+) microglia with displaced LC3 positive (+) autophagosome accumulation and autophagic vesicles (Anwar and Rivest, 2020). It is proposed that the lysosomal damage may be a key factor in inducing microglial dysfunction and poor clearance of Aβ in the late stages of AD. Further study is needed to address the relationship between lysosomal damage and autophagy due to chronic Aβ exposure.

### Phagocytosis at the Synapse

**Figure 5:**
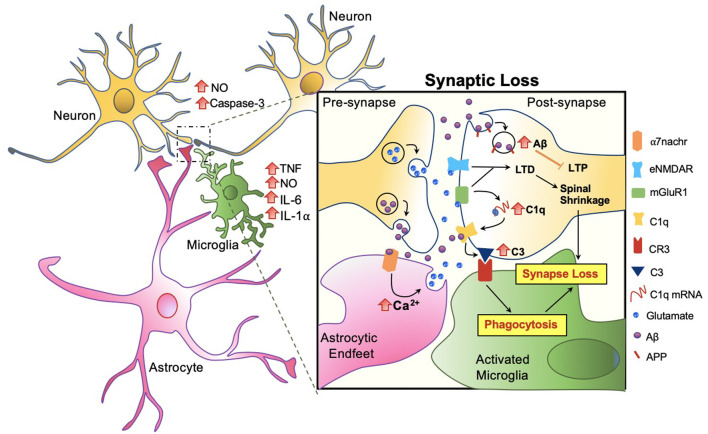
Microglia mediated synaptic loss in AD. Synaptic engulfment by activated microglia can occur through the direct effects of Aβ on synapses or indirect action *via* astrocytes. Soluble factors, tumor necrosis factor (TNF), nitric oxide (NO), interleukin-6 (IL-6), interleukin 1 alpha (IL-1α) are released by microglia, and NO and Caspase-3 by neurons, contributing towards the overall toxicity at the synapse (Rajendran and Paolicelli, 2018). In synchrony, these mechanisms result in spinal shrinkage, activated microglia, and ultimately the loss of the synapse.

Amongst the many important roles of microglia and its involvement in Aβ clearance, microglial involvement at the synaptic level in response to Aβ warrants attention. In AD patients, a significant reduction in the number of synapses has been observed, even forgoing senile plaque deposition (Cardozo et al., 2019). Studies suggest that the role of microglia in synaptic removal, normally operated during the refinement period of brain development, can be reactivated in aging or in disease. The trajectory has been best described by the complement cascade. Increased levels of the complement component 1q (C1q) and its downstream complement compound 3 (C3) at the synapse are activated, then targeted by microglia for elimination (Rajendran and Paolicelli, 2018). Recent studies show that this action occurs through tight interactions between astrocytes, microglia, and the pre and post-synapses in response to Aβ (Figure 5).

At the synapse, APP has been proposed to be an Aβ receptor which becomes internalized, increasing Aβ toxicity intracellularly and suppressing long term potentiation (LTP) in the neuron (Ripoli et al., 2014; Fá et al., 2016; Puzzo et al., 2017). Aβ also activates the complement cascade by directly binding to C1q, contributing to the upregulation of C3 which is recognized by microglial complement receptor (CR3) and triggering microglial engulfment of the synapse (Hong et al., 2016; Cardozo et al., 2019; Hemonnot et al., 2019). The complement cascade is also activated indirectly *via* astrocytes. Talantova et al. (2013) discovered that the activation of α-7 nicotinic receptor (α7nachr) by Aβ, increases intracellular Ca^2+^, and prompts glutamate release in astrocytes. Glutamate binds to metabotropic glutamate receptor 1 (mGluR1) and eNMDAR at the synapse, both of which lead to LTD and the later activating the complement cascade (Talantova et al., 2013; Cardozo et al., 2019; Figure 5). In synchrony, synaptic engulfment may be actionable when microglia and astrocytes tightly interact with pre and post-synapses, a key site previously termed the “tetrapartite synapse” (Dodds et al., 2016; Rial et al., 2016; Jay et al., 2019).

## “A Drop Hollows the Stone, Not by Force, but by Its Frequency”: Chronic Aβ Stress Leads to Brain Injury

Traditionally, the pathways of Aβ production and microglia-dependent neuroinflammation have been considered separately. However, as more studies have been performed, these processes are converging to promote understanding of neuropathology associated with AD. Recent research indicates that microglia induce continuous inflammation when Aβ levels are elevated (Webers et al., 2020). Activated microglia secrete proinflammatory cytokines that trigger a vicious and positive feedback cycle to the microglia itself and neighboring neurons, inducing persistent low-grade inflammation in the parenchyma and subsequently implementing chronic pathogenesis (Keren-Shaul et al., 2017; Webers et al., 2020). Now, it is well accepted that the neuroinflammation response is a pivotal and central player in AD pathogenesis as the third hallmark of the disease beyond Aβ plaques and fibrillary tau tangles (Webers et al., 2020).

Importantly, the chemical nature of Aβ oligomers harboring a major number of open active ends can spread easily in tissues and interact with target cells. This has shown the capacity of Aβ monomers and oligomers to penetrate, insert, or coat the plasma membrane, potentially increasing Aβ aggregations by inducing structural transitions from random coil to β sheets in Aβ1–42 peptides in neurons (Rushworth and Hooper, 2010; Drolle et al., 2014). Importantly, the dysfunction of these cellular components may lead to the activation of cellular death mechanisms and subsequent neurodegeneration observed in AD pathologies (Lansbury and Lashuel, 2006; Picone et al., 2020). In the following section, we will discuss the vicious and positive feedback mechanisms of inflammation and subsequent neurotoxicity.

### NLRP3 and ASC Specks: The Vicious Positive-Feedback Mechanism

#### NLRP3 Inflammasome

Microglia are capable of binding to soluble Aβ oligomers and fibrils *via* cell-surface receptors (CD36, CD14, and CD47) and Toll-like receptors (TLR2, 4, 6, and 9) including NACHT-, LRR-, and pyrin domain-containing protein 3 (NLRP3). Engagement of these receptors induces the release of proinflammatory cytokines and chemokines such as tumor necrosis factor (TNF) α and IL-1β, which cause sustained low-grade inflammation and neurotoxicity (Lu et al., 2008; Tejera et al., 2019; Webers et al., 2020).

**Figure 6:**
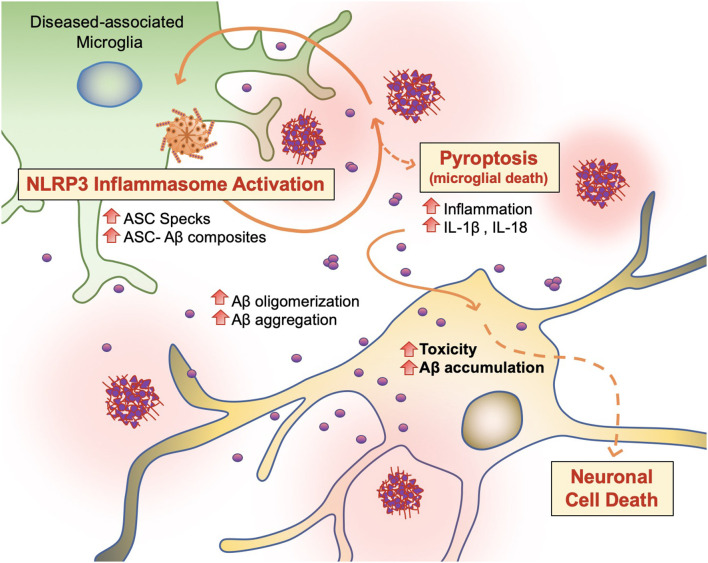
Vicious cycling of Aβ. The NLRP3 inflammasome, which behaves as an intracellular sensor for DAMP signals, is activated in diseased microglia. Specifically, in the later stages of AD, microglia activation releases ASC specks, which bind to Aβ. ASC-Aβ composites reactivate the NLRP3 inflammasome *via* a vicious cycle, prolonging inflammation and the release of inflammatory cytokines such as interleukin IL-1β and IL-18, consequently damaging neurons (Voet et al., 2019).

PAMP or DAMP signals, which trigger DAM activation and phagocytosis in microglia, also induce NOD-like receptor (NLR) family and NLRP3 inflammasome activity in microglia (Figure 6; Lučìūnaitė et al., 2019; Tejera et al., 2019). The NLRP3 inflammasome is a multiprotein complex bridged to procaspase-1 zymogen *via* the adaptor protein ASC (apoptosis-associated speck-like protein) containing a C-terminal caspase recruitment domain (CARD; Lučìūnaitė et al., 2019; Friker et al., 2020). Its assembly and activation depend on two signals: transcriptional upregulation of inflammasome components *via* the transcription factor nuclear factor κB (NF-κB), and a second signal generated by DAMP-induced ion fluxes, mitochondrial reactive oxygen species (ROS) production, or lysosomal destabilization. Indeed, both small and large Aβ molecular aggregates such as oligomers, protofibrils, and large fibrils, act as a DAMP and rapidly trigger NLRP3 (Lučìūnaitė et al., 2019).

#### ASC Specks

Following NLRP3 activation, ASC, also known as PYCARD, engages in ASC helical fibrillar assembly (Venegas et al., 2017). ASC is composed of an N-terminal pyrin domain (PYD) and a CARD. The homotypic intramolecular PYD-PYD interactions of the ASC adaptor protein initiate the formation of a helical filament, which allows intermolecular CARD-CARD interactions with the CARD domain of procaspase-1. Then, it causes the activation of mature caspase-1, which can cleave the pro-forms of inflammatory cytokines into their active forms such as IL-1B and IL-18 (Lučìūnaitė et al., 2019; Tejera et al., 2019; Friker et al., 2020; Webers et al., 2020). Interactions between ASC and caspase-1 generate large para nuclear ASC specks and release them into the intracellular space (Venegas et al., 2017; Lučìūnaitė et al., 2019; Tejera et al., 2019). ASC specks are also NLRP3 dependent and only observed in *NLRP3^−/−^* mice (Tejera et al., 2019). Released ASC or ASC specks can be functionally built into NLRP3 inflammasomes of neighboring microglia and trigger the ongoing immune response *via* microglial activity (Baroja-Mazo et al., 2014; Franklin et al., 2014; Venegas et al., 2017; Tejera et al., 2019; Friker et al., 2020).

#### ASC-Aβ Composites

Recent findings support the concept that NLRP3 inflammasome activation is connected to the seeding and spreading of Aβ pathology (Figure 6; Cho et al., 2014; Venegas et al., 2017). In AD patient brains and APP/PS1 transgenic mice, Aβ1–42 aggregates composited with ASC specks are observed rapidly after its release. Using purified ASC specks generated by immunoprecipitation and enzymatic cleavage, Aβ cross-seeding activity resulted in the acceleration of Aβ oligomerization and aggregation in injected APP/PS1 mice (Venegas et al., 2017; Friker et al., 2020) compared to ASC-deficient APP/PS1 mice. Analysis of thioflavin-T (ThT) fluorescence assays and Western blot analysis further revealed that ASC-Aβ composites are produced in a time and concentration-dependent manner (Venegas et al., 2017). Increasing evidence shows that sustained microglial activation and its peripheral inflammation increases Aβ production as a direct result of neuroinflammation. Tejera et al. (2019) found a significant increase in Aβ deposit number and size in old (15 months) APP/PS1 mice by LPS administrations but not in APP/PS1/NLRP3^−/−^ mice (Tejera et al., 2019). In this condition, ASC specks were correlatively increased. These results suggest that the NLRP-3 activation and pro-inflammation pathway affects amyloid deposition in aged APP/PS1 mice (Tejera et al., 2019).

To examine whether endogenous ASC specks contribute to this phenomenon, Venegas et al. (2017) performed direct intrahippocampal injections of ASC specks to wild-type mice and APP/PS1 mice. Administration of ASC specks increased the total number and area of Aβ immunopositive deposits compared to the contralateral injection site without changes in expression of APP or APP-cleavage products or phagocytosis. The phenomenon was absent in APP/PS1/ASC^−/−^ mice (Venegas et al., 2017). These studies suggest that an increase in ASC-Aβ composites due to the inflammatory response in microglia results in a vicious cycle between neurons and microglia, resulting in ongoing low-grade inflammation and ultimately AD progression (Figure 6; Webers et al., 2020).

#### Microglial Pyroptosis

Pyroptosis (“pyro” means fever/fire in Greek) is a unique kind of cell death by inflammatory caspases (Caspase 1, Caspase 4/5, and Caspase 11) and shows nuclear condensation, cellular swelling, and lysis. Microglial pyroptosis can be a factor in AD progression as it may release withheld Aβ plaques and ASC specks (Figure 6). In a study by Friker et al. (2020), lipopolysaccharide (LPS) primed cells were treated with ASC, Aβ, or ASC-Aβ composites. Irrespective of LPS priming, microglia exposed to ASC-Aβ composites showed a significant increase in cell death compared to ASC or Aβ alone. Interestingly, the metabolic activity in microglia treated with ASC-Aβ composites did not change within 12 h but was significantly reduced after 24 h compared to cells only treated with ASC or Aβ. ASC-Aβ composites amplified NLRP3 inflammasome activation, resulting in pyroptotic cell death. Exposure to ASC-Aβ composites amplifies the proinflammatory response, resulting in pyroptotic cell death and setting free functional ASC, and consequently inducing a vicious cycle of pathogenesis (Figure 6; Friker et al., 2020).

Caspase-1 has also been noted to be involved in microglial pyroptosis, which cleaves pyroptosis executioner protein gasdermin D (GSDMD), resulting in the formation of pores in the plasma membrane and leading to cell lysis because of ion flux and subsequent cytosolic swelling (Friker et al., 2020). When it comes to microglial pyroptosis, inflammation that results from active microglia seem to jeopardize their viability as lower molecular aggregates such as Aβ oligomers and protofibrils do not have such effects (Lučìūnaitė et al., 2019).

## Conclusion

Since the discovery of microglia types, the mechanisms for microglial activation and its possible contributions towards neuronal degeneration has become an intense topic of debate and research. A common theme amongst the players responsible for microglial activation has been its changing role on Aβ clearance depending on the stage of AD severity. It seems likely that microglial-activation is pre-programmed in homeostatic conditions or pre-clinical stages of AD, suggesting that microglia play a useful role in normal conditions and then progress into dysfunctional cells in pathological conditions as if “friends become foes” (Lee et al., 2016; Venegas et al., 2017; Tejera et al., 2019; Johnson et al., 2020). Indeed, AD pathology is through to begin 10–20 years before the first clinical manifestation, with Aβ accumulation in the cerebral spinal fluid preceding changes in the cortex (Braak and Braak, 1991; Hölttä et al., 2013; Palmqvist et al., 2016). Consistent with these observations, in both humans and AD mouse models, the absence of normally functioning microglia exacerbates Aβ pathology. On the other hand, activated microglia not only trigger inflammation but also cross-seed with neighboring neurons and astrocytes, sustaining, and accelerating diseased conditions. In combination, reconciling these contradictory functions may further uncover the role of non-cell-autonomous pathways on Aβ aggregate formation. We may find that microglia are deeply involved in the pathogenesis of AD (Anwar and Rivest, 2020; Johnson et al., 2020). In this context, future studies of the vicious cycling of Aβ aggregation *via* microglia with consideration of characterizing heterogeneous microglial types can improve our understanding of the complex pathological events in AD.

## Author Contributions

YS and HR wrote the manuscript and drew the schemes. SK, YS, and HR organized the references. JL and HR read, reviewed, and edited the manuscript. All authors contributed to the article and approved the submitted version.

## Conflict of Interest

The authors declare that the research was conducted in the absence of any commercial or financial relationships that could be construed as a potential conflict of interest.
